# Temporomandibular joint assessment in MRI images using artificial intelligence tools: where are we now? A systematic review

**DOI:** 10.1093/dmfr/twae055

**Published:** 2024-11-19

**Authors:** Mitul Manek, Ibraheem Maita, Diego Filipe Bezerra Silva, Daniela Pita de Melo, Paul W Major, Jacob L Jaremko, Fabiana T Almeida

**Affiliations:** School of Dentistry, Faculty of Medicine and Dentistry, University of Alberta, Edmonton, AB, Canada; School of Dentistry, Faculty of Medicine and Dentistry, University of Alberta, Edmonton, AB, Canada; Graduate Program in Dentistry, State University of Paraiba, Campina Grande, Brazil; College of Dentistry, University of Saskatchewan, Saskatoon, Saskatchewan, Canada; School of Dentistry, Faculty of Medicine and Dentistry, University of Alberta, Edmonton, AB, Canada; Radiology and Diagnostic Imaging, Faculty of Medicine and Dentistry, University of Alberta, Edmonton, AB, Canada; School of Dentistry, Faculty of Medicine and Dentistry, University of Alberta, Edmonton, AB, Canada

**Keywords:** temporomandibular joint disc, magnetic resonance imaging, artificial intelligence, systematic review

## Abstract

**Objectives:**

To summarize the current evidence on the performance of artificial intelligence (AI) algorithms for the temporomandibular joint (TMJ) disc assessment and TMJ internal derangement diagnosis in magnetic resonance imaging (MRI) images.

**Methods:**

Studies were gathered by searching 5 electronic databases and partial grey literature up to May 27, 2024. Studies in humans using AI algorithms to detect or diagnose internal derangements in MRI images were included. The methodological quality of the studies was evaluated using the Quality Assessment Tool for Diagnostic of Accuracy Studies-2 (QUADAS-2) and a proposed checklist for dental AI studies.

**Results:**

Thirteen studies were included in this systematic review. Most of the studies assessed disc position. One study assessed disc perforation. A high heterogeneity related to the patient selection domain was found between the studies. The studies used a variety of AI approaches and performance metrics with CNN-based models being the most used. A high performance of AI models compared to humans was reported with accuracy ranging from 70% to 99%.

**Conclusions:**

The integration of AI, particularly deep learning, in TMJ MRI, shows promising results as a diagnostic-assistance tool to segment TMJ structures and classify disc position. Further studies exploring more diverse and multicentre data will improve the validity and generalizability of the models before being implemented in clinical practice.

## Introduction

Temporomandibular disorder (TMD) is an umbrella term for structure and/or functional disorders that affect the TMJ, masticatory muscles, and associated structures. These disorders may present with clinical signs and symptoms such as pain in the face and preauricular area, articular noises, and limitation in opening and closing mouth.^1^ A recent systematic review reported a TMD prevalence of 31% in the general population with disc displacement being the most common manifestation.^2^

The most accepted diagnostic criteria (DC/TMD) for TMD diagnosis is based on clinical examination supported by diagnostic imaging.^1^ This guideline considers magnetic resonance imaging (MRI), as the reference standard to assess the soft tissue structures of the joint. MRI is a non-invasive, non-ionizing radiation imaging technique which due to its superior soft tissue contrast provides great information on disc shape and position and proper diagnosis of TMJ internal derangement. However, the interpretation of TMJ in MRI images is a challenging task and requires a high-level of expertise.^3^ Considering that a precise diagnosis is essential for effective treatment planning, new technologies and approaches including imaging fusion, segmentation and classification using artificial intelligence (AI) have been proposed to assist and improve TMJ assessment in MRI images.^4–6^

AI has been used to describe algorithms that can think and reason rationally like humans imitating mental activity processes. It is often defined as a non-biological ability to solve complex tasks by analysing large datasets and making inferences and decisions using statistical methods. While it seems to mimic rational human thought, it actually learns patterns from data, operating in a “black box” manner that obscures the intermediate reasoning process.^7^ Machine learning (ML) is a form of AI that enables computer systems to learn patterns from a large amount of data.^8^ It is an approach largely used in automated medical imaging analysis to predict the diagnosis of different diseases, intending to maximize the diagnosis process and consequently, improve therapeutic decisions.^9^

AI algorithms have been applied in the diagnostic imaging of TMD in different imaging modalities.^6^^,^^10^ A recent comprehensive review of the literature showed a growing interest in employing AI models for TMD with studies focusing on TMJ osteoarthritis, TMJ internal derangements, and Juvenile Idiopathic Arthritis.^6^ In addition, a systematic review of the literature identified a variety of AI models developed to assess TMJ osteoarthritis in cone beam computed tomography (CBCT) and panoramic images with performance ranging from good to moderate.^10^ Therefore, the objective of the current study was to investigate the application of AI algorithms for the assessment of TMJ structures and the diagnosis of TMJ internal derangement in MRI images through a systematic review of the literature.

## Methods

The systematic review was reported following the recommendations of the “Preferred Reporting Items for Systematic Reviews and Meta-Analyses Protocols (PRISMA) Statement”.^11^ The review was registered at the Open Science Framework and can be found at the following address: https://osf.io/5b3dp/

### Eligibility criteria

This systematic review addressed the following question “What is the diagnostic capability of AI algorithms to identify TMJ articular disc and/or to diagnose TMJ internal derangements in MRI images? The research question was formatted using the following PIRDS framework: Population (P): patients with or without TMD; Index test (I): AI algorithms trained in MRI images for visualization of TMJ articular disc of TMJ internal derangement diagnosis; Reference standard (R): human interpretation; Diagnosis (D): performance of the index test for the detection of TMJ articular disc or diagnosis of TMJ internal derangement (disc displacement, disc degeneration, disc perforation). Types of Studies included (S) Observational Studies (Diagnostic tests involving artificial intelligence, machine learning, or deep learning algorithms that were compared with human radiographic interpretation).

The exclusion criteria were the following: (1) Studies using clinical data but no imaging; (2) Studies in which AI was not used or it was used with another focus other than TMJ detection and diagnosis; (3) Studies training AI with an imaging modality other than MRI; (4) Studies with no reference standard (human labelling) as a comparison; (5) Reviews, personal opinions, abstracts, case reports, cases series, book chapters, letters.

### Information sources and search strategy

A structured electronic search was performed in the following databases: Medline, Embase, Scopus, and Web of Science without limiting the date of the publication. The grey literature was accessed using Google Scholar by screening the abstracts for the first 100 results (filtered by “relevance”). The search across all databases was carried out in August 2023 and updated on May 27, 2024. Original studies published in peer-reviewed journals in English were considered. The search strategy applied to each database is available in Appendix S1.

In addition to the electronic search, experts’ consultations and hand searches of the reference lists of the selected articles screened were implemented. All references were managed by Covidence (Covidence systematic review software, Veritas health innovation, available at www.covidence.org) where duplicate papers were removed.

### Study selection and data collection process

A two-phase selection of articles was conducted. In Phase 1, 2 authors (M.M and I.M) reviewed the titles and abstracts of all the references independently. These authors selected articles that appeared to meet the inclusion criteria based on their titles and abstracts. In Phase 2, the same authors assessed the full text of all screened articles and excluded studies that did not meet the inclusion criteria. Disagreements between the 2 authors were initially resolved by a third reviewer (F.T.A). The final selections were always based on the full text of the publication. Covidence was used in this process and blindness was ensured.

Two reviewers (M.M and I.M) extracted the following information from the included studies: study characteristics (authors, country/year, study aim), sample characteristics (population, sample size, data set information), index test information (AI algorithm used, training/testing/validation dataset, images used, extracted information from the images), reference standard (ground truth approach), main outcomes related to the AI performance and conclusions. The extracted information was checked by 2 other authors (D.F.B.S and F.T.A). Performance metrics such as sensitivity, specificity, AUC, dice coefficient, and Hausdorff distance were the main metrics considered as outcome measures. Prediction time information was also extracted when available.

### Risk-of-bias assessment

To assess the methodological quality and applicability of the included studies, the Quality Assessment Tool for Diagnostic Accuracy Studies-2 (QUADAS-2) was applied. Two reviewers (M.M and F.T.A) independently evaluated the quality of each included study and scored each item as “yes”, “no” or “unclear”. The risk was judged as Low (L), High (H), or unclear (U). A third reviewer (D.F.B.S) was involved when disagreements arose. Furthermore, a checklist for AI studies in dental research was applied in the included studies.^12^ This tool involves several factors such as data (sampling, processing, and protection), sample size, reference test, clustering, test dataset (model and training), and computational resources. Each factor’s items were assessed as present (1) or absent (0), and the values were tabulated and analysed. The checklist was independently applied by 2 reviewers (M.M and D.F.B.S). In cases of disagreement, a third reviewer (F.T.A) was consulted to reach a consensus.

## Results

### Study selection

**Figure 1.**
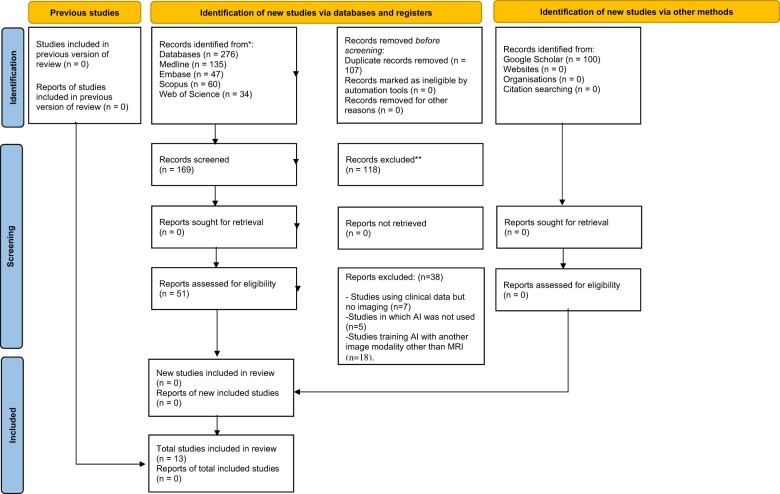
Flowchart diagram.

A flow diagram detailing the process of identification, inclusion, and exclusion of the studies is shown in Figure 1. The database search retrieved a total of 276 articles. One hundred and seven duplicates were removed and the titles and abstracts of the remaining 169 studies were screened in the first phase of the review. After reading the titles and abstracts, 118 studies were excluded. In the second phase of the review, the full texts of 51 studies were checked and 38 were excluded due to the following reasons: Studies using clinical data but no imaging (*n* = 7), studies that did not use AI (*n* = 5), studies that used another imaging modality (*n* = 18), studies that did not use a reference standard as a comparison (*n* = 2), and wrong study design (*n* = 6) (Appendix S2). Finally, 13 studies satisfied the inclusion criteria of this review and were selected for the qualitative synthesis.^13–25^

### Study characteristics

The included studies were published from 2021 to 2023. All articles were written in English. The studies were conducted in 6 different countries: China,^13^^,^^19^^,^^21^^,^^23^ Japan,^14^^,^^20^^,^^25^ Korea,^16^^,^^17^^,^^24^ Taiwan,^15^ Turkey,^22^ and Canada.^18^

The sample size ranged from 20 to 1260 patients, including healthy or those with alterations in any of the TMJ components, with ages ranging from 13 to 74 years. All studies had at least 1 expert to analyse the images serving as the ground truth to train the AI models. Most studies evaluated the position of the articular disc, with or without displacement.^13–15^^,^^17^^,^^19–25^ In only 1 study, disc perforation was analysed,^16^ while 2 other studies investigated cases of condylar degeneration compared to the position of the articular disc (normal or displaced).^21^^,^^22^ Finally, Wu et al.^23^ investigated cases of articular disc displacement and joint deformation; and Li et al.^18^ evaluated the normal anatomy of the TMJ components. A summary of the descriptive characteristics of the included article is given in Tables 1 and 2.

**Table 1.** Summary of results: study/sample characteristics and dataset characteristics from the included studies.

Study/sample characteristics			Dataset characteristics		
Author, year, country	Population/sample/age range/sex	Study objective/target	Data used	Reference standard	Algorithms/computer systems used
Bai et al.^13^, China	600 patients Shanghai Ninth People’s Hospital (1189 TMJs -577 with DD and 612 healthy joints); age/sex NA	To determine whether the AI-assisted method could increase dentists’ ability to diagnose ADD in TMJ MRIs.	MRI (3.0 T) Sequence/plane NA	Two radiologists and a TMJ specialist	TMJ MRI-Net (Multimodal Stepped Attention Net)
Ito et al.^14^, Japan	10 ADD patients (106 images), 19 to 39 years (8F; 2M); 10 normal disc patients (111 images), age 18 to 41 years (8F; 2M)	To construct DL segmentation algorithms for automatic detection and segmentation of TMJ disc.	MRI (3.0 T) PD sagittal	Two orthodontists and an OMR	3DiscNet; U-Net SegNet-Basic
Kao et al.^15^, Taiwan	52 DD patients (195 images) and 32 with normal discs (105 images); age 20+	To propose a new diagnostic tool for automatically extracting discriminative features and detecting TMJ DD with AI.	MRI (1.5 T) T1 coronal; T2 and PD oblique sagittal	One OMS	InceptionResNetV2, InceptionV3, DenseNet169, and VGG16
Kim et al.^16^, Korea	289 patients (168 non-perforated joints, 22-33 years, and 131 perforated joints, 26.5-44.5 years); 40 M joints and 258 F joints	To develop a DL-based algorithm to predict TMJ disc perforation based on the findings of MRI.	MRI (3.0 T) T1 and T2 sagittal	Two OMS and an OMR	Multilayer perceptron (MLP), Random forest, Disc Shape Alone
Lee et al.^17^, Korea	1260 patients with DD (2520 TMJs—2051 bilateral images and 468 images); 861M, 399F)	To investigate the usefulness of DL-based automatic detection of ADD from MRI of patients with TMD.	MRI (3.0 T) T1, T2 and PD sagittal oblique images	One TMD specialist	VGG16 CNN
Li et al.^18^, Canada	140 patients (280 TMJs, 2614 images) - normal and displaced discs; age over 18 years	To employ two DL approaches to delineate the condyle, articular eminence and to automatically detect the disc	MRI (1.5 and 3.0 T) PD sagittal images	One OMR	UNet++ nnU-Net
Lin et al.^19^, China	507 patients (1014 TMJ, 9009 images); 426F, 81M; age over 16 years	To develop a model to assist clinicians in evaluating ADD before orthodontics treatment.	MRI (1.5 T) T1 and T2 sagittal and axial corrected coronal images	Three physicians	CNN (RestNet and ImageNet)
Nozawa et al.^20^, Japan	357 patients (600 TMJ, 1200 images); age/sex NA	To construct a DL model for automatic segmentation of the TMJ disc, and to evaluate the performances using internal and external test data.	MRI (0.4 and 3 T) PD, T1 and T2 sagittal	Two radiologists	Modified UNet CNN
Orhan et al.^21^, China	107 patients (214 TMJ normal and ADD) 34M, 73F; 9-74 years	To propose a ML model and assess its ability to classify TMJ pathologies on MRI.	MRI (1.5 T) T1, T2 and PD sagittal/coronal	Two radiologists	LR, random forest, decision tree, KNN, XGBoost, and SVM
Ozsari et al.^22^, Turkey	200 patients (2576 images) (with and without TMD); age/sex NA	To interpret TMJ disorders displayed on MRI by using DL approaches and to assess its effectiveness.	MRI (1.5 T) T1, T2, MERGE, and PD sagittal	Two OMR	Xception, ResNet-101, MobileNet, InceptionV, DenseNet-121, ConvNeXt, ViT
Wu et al.^23^, China	204 TMJs with DD and/or deformation; age/sex NA	To evaluate four DL semantic segmentation methods to support the diagnosis of TMJ disease on MRI	MRI (3.0 T) Sequence/plane NA	An OMR and a radiologist	Unet, Attention-Unet, Unet++ and C2FTrans
Yoon et al.^24^, Korea	728 participants (1195 TMJ, 2390 images; normal and ADD); 15 to 77 yrs	To propose a clinical decision support engine that diagnoses TMJ ADD using MRI.	MRI (1.5/3.0T) Sequence/plane NA	Four specialists and three residents	RetinaNet ImageNet RestNet50
Yoshimi et al.^25^, Japan	49 normal and DD patients (536 images), 13-45 yrs, 36F/13M	To evaluate the robustness of DL-based ED-CNNs for segmenting TMJ discs	MRI (3.0 T) PD oblique sagittal/coronal; T2 sagittal	Two orthodontists and two OMR	ED-CNN model with CLAHE

Abbreviations: ADD = anterior disc displacement; AI = artificial intelligence; CLAHE = contrast-limited adaptive histogram equalization; DD = disc displacement; DL = deep learning; ED-CNNs = encoder decoder convolutional neural networks; F = female; KNN = k-nearest neighbours; LR = logistic regression; M = male; MERGE = multiple echo recombined gradient echo; ML = machine learning; MRI = magnetic resonance imaging; OMR = oral and maxillofacial radiologist; OMS = oral and maxillofacial surgeon; PD = proton density; T = tesla; TMJ = temporomandibular joint; TMD = temporomandibular disorders; SVM = support vector machine

**Table 2.** Summary of results continuing**:** dataset characteristics continuing, main results and conclusions from the studies included.

Author/year	Test/Training/Validation sample	Training Dataset Features	Main Results/performance	Main conclusions
Bai et al.^13^	Training = 960 TMJs (7680 images) Testing = 229 TMJs (1832 images)	Extracted features from unidirectional spatial sparse MRI data and generated auxiliary diagnosis results of TMJ DD and/or its reducibility.	DD physician with AI assistance—AUC 0.99 (95 % CI: 0.99-1.00), statistically different from physician without AI (AUC 0.92 95 % CI: 0.90-0.95). AUC for DD with/without reduction for the physician with AI was 0.95 (95 % CI: 0.92-0.98), while the AUC for physician only was 0.82 (95 % CI: 0.75, 0.89) (*P* < .0001).	The AI-assisted strategy improved the diagnostic accuracy of physicians (Orthodontist and General Dentistry) on ADD in TMJ MRI.
Ito et al.^14^	Training/Testing (normal position of AD) = 88 images/23 images; Training/testing (DD) = 84 images/22 images; Training/testing (normal and DD) = 173 images/44 images	ROIs around the articular discs were extracted from the datasets.	U-Net showed lower values 3DiscNet and SegNet-Basic (one-way ANOVA with Tukey HSD; *P* < .001) for all three metrics (DC, SN and PPV) for normal position (DC, 0.45 ± 0.10; sensitivity 0.40 ± 0.11; PPV 0.55 ± 0.16) and displaced discs (DC 0.33 ± 0.21; SN 0.28 ± 0.20; PPV 0.44 ± 0.24).	3DiscNet and SegNet-Basic trained on manually segmented MRI can segment TMJ disc on MRI images.
Kao et al.^15^	Training ∼ 1.28 million images Testing = 300 deidentified MRI scans	Using image classification with localization, a supervised algorithm was trained to predict classes by applying masking around the object in the image.	InceptionResNetV2—F1 score ranging from 83 to 93%; VGG16—F1 score ranging from 68 to 86%; InceptionV3 (F1 score: 0.9) had superior performance on the test set compared with the other models (F1 scores: DenseNet169, 0.89; InceptionResNetV2, 0.86; VGG16, 0.73).	Automated detection of TMJ DD from MRI images is a promising technique that involves using DL networks. It can be used to support clinicians in diagnosing TMJ DD.
Kim et al.^16^	Training = 80% Validation = 20%	Data containing features extracted of MRI images were applied to build and validate prediction models using RF and MLP techniques, the latter using the Keras framework.	MLP produced the highest performance (AUC 0.94); MLP model showed SN 0.85 and SP of 84.8%. Random forest model showed SN of 0.96 and SP of 0.75.	Implementing deep learning showed superior performance in predicting disc perforation in TMJ compared to conventional methods and previous reports.
Lee et al.^17^	Training = 1640 images Validation = 411 images Testing = 468 images	Data augmentation and the Adam optimizer were applied to reduce the risk of overfitting the DL model.	VGG16 CNN model: accuracy 0.83, SN 0.821, and SP 0.845.	Using pre-trained weights not only improved the prediction accuracy but also clarified Grad-CAM images by deactivating uninteresting gradient values.
Li et al.^18^	Training/testing: 1898 and 716 images respectively	The data were labelled using an in-house developed software implemented based on VTK and PyQt5.	2D: UNet++ DC of 0.70 for the articular disc and 0.93 for the condyle, with an average HD of 0.88 mm for the articular eminence, vs. 0.70, 0.94 and 0.83 mm for the nnU-Net, respectively. 3D: Both networks performed similarly 0.68, 0.92 and 2.66 mm for UNet++ and 0.70, 0.93 and 2.21 mm for nnU-Net, respectively	Both networks achieved near-expert performance for the segmentation of all three TMJ structures on MRI. The models performed better than novice readers while delineating the structures
Lin et al.^19^	Close mouth position Training/testing/validation: 3,452 images/432 images/431 images, respectively Open mouth position Training/testing/validation: 3756 images/469 images/469 images	The images were selected in which condyle was visible and located within 1/3 of the centre of the image. The pixel coordinates were taken (160,160) as the centre point, intercept the 200*200 pixels image, and saved them in JPEG format.	For the maximum open mouth position model had accuracy and AUC of 0.970 (±0.007) and 0.990 (±0.005), respectively. For closed mouth position models, the accuracy and AUC of diagnostic Criteria 1 were 0.863 (±0.008) and 0.922 (±0.009), respectively significantly higher than that of diagnostic Criteria 2 with 0.839 (±0.013) (*P* = .009) and AUC of 0.885 (±0.018) (*P* = .003).	The models could be used to assist clinicians in assessing TMJ disc before orthodontic treatment, and hence reduce the occurrence of serious treatment complications.
Nozawa et al.^20^	Training and Validation = 800 images Testing = 200 images External validation = 200 images	This convolutional neural network consisted of a convolutional layer, a rectified linear unit activation function layer and a pooling layer.	Recall (sensitivity) of 0.92 and precision of 0.82 for ADD; 0.91 and 0.90 for normal disc position respectively	The DL segmentation used for disc identification yielded high performance. The model created in the study may aid in identifying disc position on MRI
Orhan et al.^21^	Training = 80% of volume of interest of the images Validation = 20% of volume of interest of the images	texture features containing grey-level cooccurrence matrix (GLCM), grey-level run length matrix (GLRLM), and grey-level size zone matrix (GLSZM) were used.	The AUC, sensitivity, and specificity for the training set were 0.89 and 1, while those for the testing set were 0.77 and 0.74, respectively, for condylar changes and disc displacement, respectively.	The ML model can classify the condylar changes and TMJ DD. This study also demonstrated that the combination of specific MRI based radiomic features with image variables can predict TMJ pathologies.
Ozsari et al.^22^	Training/Testing = 80%/20% Validation = 20% of the training data set	Screenshots were taken for articular disc position, for effusion, and condylar degeneration.	The ICC values of models were 0.58 for accuracy, 0.57 for precision, 0.63 for sensitivity, 0.64 for F1-score, 0.28 for NPV, 0.79 for specificity, 0.74 for AUC and 0.67 for kappa score. While the correlation between models for the NPV value was low, the results for other metrics were found to be acceptable.	Whether learning was carried out in part related to the disease with Grad-CAM images. Considering the results, the reliability between architectures was interpreted as satisfactory.
Wu et al.^23^	Training/Testing Set =80%/20%	The final segmentation is performed by combining the results of coarse region localization (i.e. CGT), boundary identification (i.e. BLT) and boundary refinement (i.e. CNN).	C2Ftrans model performs best with the highest dice of 73.5% and the lowest computational complexity.	C2Ftrans model performs best with the highest dice of 73.5% and the lowest computational complexity.
Yoon et al.^24^	Training/testing and external testing/validation: 80% of 1430 images (715 TMJ)/360 images (180 TMJ)/600 images (300 TMJ) 20% of 1430 images (715 TMJ), respectively	In the low-contrast ROI MRI images predicted from the ROI detection model. CLAHE divided the image into smaller grids and applied equalization within the grid, distributing the intensity of pixels that exceed the clip limit evenly among other pixels	The ROI detection model achieved precision of 0.819 at 0.75 IOU thresholds in the internal test. In internal and external tests, the ADD classification model achieved AUC characteristic values of 0.98 and 0.96, SN of 0.95 and 0.92, and SP of 0.919 and 0.89, respectively.	The proposed explain- able DL-based engine provides clinicians with the predictive result and its visualized rationale.
Yoshimi et al.^25^	Experiment 1 Training/testing: 430/106 images respectively Experiment 2 Training/testing : 437/99 images, respectively	ED-CNN model, which was trained using an Adam optimizer with a learning rate of 2.0 × 10^−3^.	The values of 3 metrics—DC, SN and PPV- were significantly improved when images were preprocessed with CLAHE (*P* < .05).	The proposed system provides promising results for a DL-based, fully automated segmentation method for TMJ articular disks on MRI.

Abbreviations: AD = articular disc; AUC = area under the curve; DD = disc displacement; ADD = anterior disc displacement; AI = artificial intelligence; CLAHE = contrast-limited adaptive histogram equalization; DC = dice coefficient; DD = disc displacement; DL = deep learning; ED-CNN = convolutional neural network; F = female; HD = Hausdorff distance; KNN = k-nearest neighbours; LR = logistic regression; M = male; MERGE = multiple echo recombined gradient echo; ML = machine learning; MLP = multilayer perceptron; MRI = magnetic resonance imaging; OMR = oral and maxillofacial radiologist; OMS = oral and maxillofacial surgeon; PD = proton density; PPV = predictive positive value; SN = sensitivity; SP = specificity; T = tesla; TMJ = temporomandibular joint; TMD = temporomandibular disorders; RF = random forest; SVM = support vector machine

### RoB and applicability

**Figure 2.**
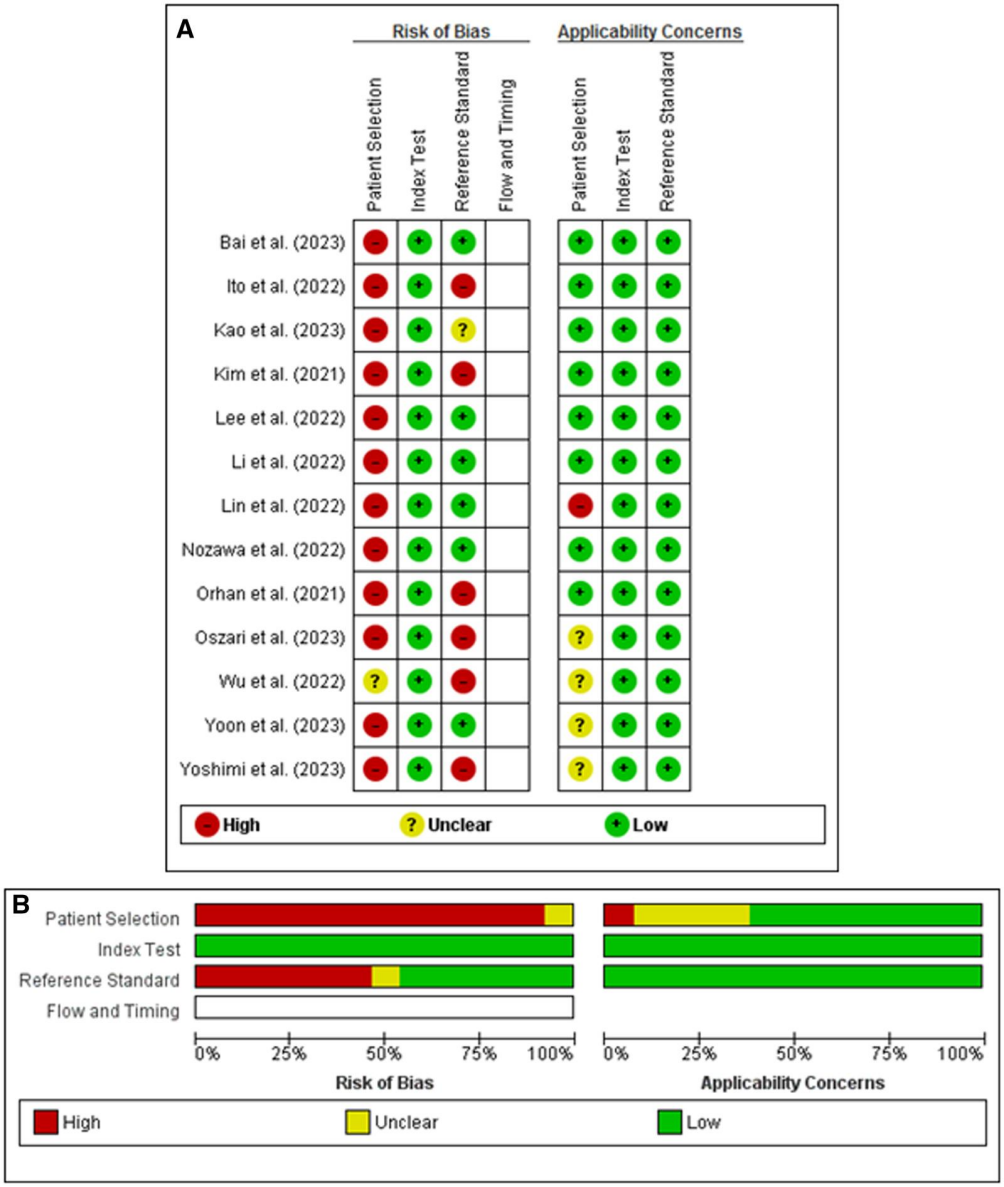
QUADAS-2 data for quality assessment including risk of bias and applicability concerns of the included studies that evaluated AI for identification of TMJ structures and TMD in MRI. (A) Individual studies, (B) Within studies.

None of the studies fulfilled all the methodological quality criteria. The Risk of bias (RoB) of patient selection was scored as “unclear” for one study,^23^ while the rest were considered “high risk”.^13–22^^,^^24^^,^^25^ Six studies were assessed as “low risk”,^13^^,^^17–20^^,^^24^ another 6 as “high risk”^14^^,^^16^^,^^21–23^^,^^25^ and 1 study was assessed as “unclear”^15^ in reference standard RoB domain. All studies presented low applicability concerns in index test and reference standard domains. On the other hand, in the domain of patient selection, 4 studies were evaluated as “unclear”^22–25^ and 1 study as “high risk”.^19^  Figure 2 shows the QUADAS-2 criteria for each included study.

The results for the dental research AI checklist are presented in Appendix S3. Notably, “clustering” was only presented in 1 of the studies.^21^ Most studies reported “sample size”.^13^^,^^14^^,^^15^^,^^17^^,^^18^^,^^20–22^^,^^24^^,^^25^ Six studies reported “protection”.^15^^,^^16^^,^^18^^,^^19^^,^^21^^,^^24^ However, all studies addressed “processing”, “reference test”, “dataset” and “computational resource”. Only 1 study did not present “sampling”.^23^

### Results of individual studies

#### Disc displacement

Most studies investigated the performance of AI to assess disc position.^13–15^^,^^17^^,^^19^^,^^20–25^ In the study by Bai et al.,^13^ with TMJ MRI-Net system, the AUC of disc displacement for the physician with AI assistance group was 0.995 (95% CI: 0.990-1.000), which was statistically different from the AUC for the physician without AI assistance group, which equalled 0.926 (95%CI: 0.900-0.953). The AUC of patients for whether the disc displacement is with/without reduction for the physician with AI assistance group was 0.954 (95%CI: 0.924-0.984), while the AUC for the physician without AI assistance group was 0.827 (95%CI: 0.759, 0.894), and the difference between the assessments with and without AI assistance was statistically significant (*P* < .0001).

Kao et al.^15^ assessed the detection of TMJ disc displacement using 4 CNN architectures (InceptionResNetV2, InceptionV3, DenseNet169, and VGG16) and found that InceptionResNetV2 was the best-performing deep learning classifier, reaching an accuracy of 100% when data augmentation was used during training. However, InceptionV3 (F1 score: 0.9) had superior performance on the test set compared with the three other models (F1 scores: DenseNet169, 0.89; InceptionResNetV2, 0.86; VGG16, 0.73).

Nozawa et al.^20^ used only the U-Net model to assess disc displacement, obtaining a recall of 92.9% and precision of 82.6% for anterior disc displacement detection, and a recall of 91.6% and precision of 90.2% for normal disc position detection. Ito et al.^14^ also used the U-Net model in addition to 3DiscNet and SegNet-Basic to automatically detect and segment the TMJ disc in MRI. In this study, 3DiscNet and SegNet-Basic models showed better performance than the U-Net model with similar dice coefficient values of 0.76 and 0.72 for normal discs and 0.70 and 0.68 for displaced discs respectively.

Lin et al.^19^ developed deep learning models (Resnet and ImageNet) using CNN to automatically detect anterior disc displacement using MRI. Two diagnostic criteria were evaluated for the closed mouth position. Criteria 1 classified disc position as normal, intermediate, and full anterior disc displacement. Criteria 2 classified disc position as normal and displaced. The disc position for the open mouth was classified as normal and disc displacement. The performance of the maximum open mouth position model had 0.970 accuracy, 0.98 sensitivity, 0.95 specificity, and an area under the curve (AUC) of 0.99. The closed mouth diagnostic Criteria 1 model had 0.86 accuracy, 0.73 sensitivity, 0.92 specificity, and 0.922 AUC. The closed mouth diagnostic Criteria 2 had 0.86 accuracy, 0.67 sensitivity, 0.91 specificity, and 0.89 AUC. The accuracy and AUC of diagnostic Criteria 1 were significantly higher than that of diagnostic Criteria 2 (*P* < .01).

Similarly, Yoon et al.^24^ also used the ImageNet model, but with 2 other models, RetinaNet and resnet50 to create heat maps for disc displacement detection. In their study, the region of interest detection model achieved a mean average precision of 0.81 at 0.75 intersection over union thresholds in the internal test. In internal and external tests, the anterior disc displacement classification model achieved AUC values of 0.98 and 0.96, sensitivities of 0.95 and 0.92, and specificities of 0.91 and 0.89, respectively.

In the study by Ozsari et al.,^22^ 7 different networks (a basic CNN, and the following fine-tuned pre-trained CNN: Xception, ResNet-101, MobileNetV2, InceptionV3, DenseNet-121, and ConvNeXt) for closed mouth disc position were assessed. The best model was MobileNetV2 with an accuracy of 0.97 followed by ResNet-101 with an accuracy of 0.91.

Yoshimi et al.^25^ developed a CNN model for segmenting TMJ discs using original images acquired from 2 3 T MRI machines. In addition, they examined the impact of contrast-limited adaptive histogram equalization (CLAHE), a typical preprocessing method for imaging enhancement, on the performance of the model. The values of 3 metrics—dice similarity coefficient value, sensitivity, and positive predictive value- were significantly improved when images were preprocessed with CLAHE (*P* < .05). Lee et al.^17^ used the VGG16 CNN model and achieved an accuracy of 0.83 (sensitivity of 0.82 and specificity 0.84). The study by Wu et al.^23^ evaluated articular disc displacement and joint deformation comparing Unet, Attention-Unet, Unet++, and C2FTrans for data segmentation and showed that the C2Ftrans model performs best with the highest dice of 73.5% and the lowest computational complexity.

The study of Ozsari et al.^22^ used different deep learning approaches to assess TMJ disorders (joint effusion, condyle degeneration, and disc displacement) and compared their performance. For disc position, MobileNet V2 and ResNet-101 presented the highest performance in closed (AUC 0.95) and open-mouth (AUC 0.75), respectively. The authors found that MobileNet V2 had the most effective architecture for all TMJ disorders.

One study^21^ used radiomic features to develop models to assess TMJ disc displacement and condylar degenerative changes in MRI images. A radiomics platform was used to extract 90 radiomic features, mostly under shape and texture classifications, from segmented TMJs discs classified as normal, anterior displacement with and without reduction. Fifty-six radiomic features were identified as associated with TMJ pathologies and had significant performance in differentiating between normal and displaced discs. Random forest classifier was the best method for disc displacement detection with a precision/recall/F1 score of 0.99 in the training dataset and 0.79/1.00/0.88 in the testing dataset, respectively.

#### Disc perforation

Only 1 study used deep learning models to predict TMJ disc perforation.^16^ In this study, the authors used a sample of 299 perforated and non-perforated discs based on the surgery results to train and validate the model. Human data extracted from disc shape, bone marrow signal, joint space, disc/condyle relationship, and changes of condyle and fossa from T1 and T2 sagittal images were used to build the disc perforation prediction model. Random forest and multilayer perceptron (MLP) techniques were used. MLP had the best performance (AUC 0.94; 95% CI 0.88-0.99), followed by random forest (AUC 0.91; 95% CI 0.84-0.98).

#### TMJ anatomy identification

One study exclusively evaluated the performance of 2 deep learning models (nnU-Net and UNet++) to segment TMJ anatomical structures (disc, condyle, and eminence), in 2D images and 3D volumes.^18^ In this study, in 2D, the UNet++ model achieved an average dice coefficient of 0.70 for the articular disc and 0.93 for the mandibular condyle, with an average Hounsfield distance of 0.88 mm for the articular eminence, vs. 0.70, 0.94 and 0.83 mm for the nnU-Net, respectively. Both networks performed similarly in 3D: 0.68, 0.92, and 2.66 mm for UNet++ and 0.70, 0.93, and 2.21 mm for nnU-Net. In this study, the centroid disc was a new metric proposed to assess disc position. The models identified the disc centroid within 1.1 mm which was intermediate in performance between human experts (0.6-0.8 mm) and non-experts (1.9 mm). Both models generated expert-level performance for all three TMJ anatomical structures segmentation.

#### Prediction time of the models

Only 2 studies reported the prediction time of the models. Li et al.^18^ reported a prediction time ranging from 0.6 to 18 s and Bai et al.^13^ model prediction ranged from 16.4 to 27.4 s. Ozsari et al.^22^ mentioned it reduces the diagnosis time, but no numbers were reported.

## Discussion

This systematic review assessed the performance of AI models in the identification of TMJ discs and the diagnosis of disc internal derangements in MRI images. AI algorithms showed promising results for the tasks of detecting TMJ disc, condyle, and articular eminence as well as classifying disc position with performance metrics representing accuracy ranging between 0.70 and 0.99 compared to human experts. A meta-analysis was not possible due to the high heterogeneity of AI models employed and the dataset characteristics among the included studies.

All included studies employed CNNs based on a deep learning approach in their models, a subset of ML. CNN architecture incorporates a sequence of convolutional layers to process image inputs while learning higher-level imaging features.^8^ CNN algorithms have become tools of great interest for automated image analysis and diagnosis due to their ability to minimize the likelihood of human errors and aid in performing complex visual tasks by matching or even surpassing human capabilities. This technology has been employed in dental image diagnosis as a diagnostic assistance tool to help clinicians and novice radiologists in interpreting images more effectively.^26^ In addition, all included studies used a supervised learning approach to train the models using labelled datasets. Weakly-supervised or unsupervised techniques, an emerging technology, were not used.

Many of the included studies used 1^15^^,^^17^^,^^18^ or 2 experts^20–23^ to label the training dataset of the proposed models. When more than 1 expert was used, there was limited information regarding the calibration process between them. No external reference standard besides MRI is available. This type of ground truth can generate an ambiguous gold standard for the model, with imaging artifacts and human expert biases related to personal experiences incorporated into the algorithms, lending potential bias to the results of the studies.

All the studies used localized available samples from single centres. Most of the studies included cases with normal and anterior displaced discs,^13–15^^,^^17^^,^^19–25^ and only 1 study assessed disc perforation (AUC 0.94).^16^ There was no data on disc degeneration or fragmented discs. Twelve of the 13 studies were developed in Asian countries. These types of single-centre models are prone to over-fitting and are likely to apply only to the population on which they were trained. The development of robust clinical AI models depends not only on access to high-volume but also on high-quality data. More diverse data is desirable to improve the robustness of the algorithms. Further studies using multi-centre data are needed to improve confidence in the generalizability and the external validation of the AI models to different populations.

In the AI MRI-based models, another layer of heterogeneity is introduced in the dataset in terms of (1) the MRI sequence protocol (T1, T2, proton-density, or any others) and (2) the orientation planes used to train the model (sagittal, coronal, or axial). T2 and proton-density are the sequences recommended to assess TMJ internal derangement with the sagittal view as the best plan to assess disc position.^27^ Eight studies used more than 1 sequence protocol and orientation plane to develop their model.^16^^,^^17^^,^^19–22^^,^^25^ Two studies used only PD sagittal images^14^^,^^15^^,^^18^ and 3 studies didn’t provide information about sequences and planes used to develop the model to diagnose disc displacement.^13^^,^^23^^,^^24^ Since AI relies on high-quality data, these technical parameters are important to consider when developing the models.

Regarding the data used as input to train the models, most of the studies used 2D slices from MRI images to train the models slice by slice. Interestingly, 1 study using both 3D volume and 2D slices as input found better results for segmenting the disc and identifying disc position in nnUnet model using 3D. The authors hypothesized that the enhanced results from the 3D model were due to the segmentation of 3D volumes, which enables the model to make predictions by leveraging information from adjacent slices, similar to the way human readers assess the images.^18^ The ability to visualize the disc and its surrounding structures in 3D as a volume allows for comprehensive tissue visualization across all directions and slices, which, provides a more representative image of the TMJ disc that can facilitate clinical decision-making. That would be an interesting topic to be further explored in future studies.

The purpose of automating TMJ disc evaluation in MRI images is not only to improve the diagnosis but also to reduce the diagnosis time for the clinician, improving workflow and hence making the delivery of care more efficient. Therefore, prediction time is an important item to be reported when assessing the performance of a model to be implemented in the clinic. Only 1 study^18^ reported the prediction time of the model. Future studies should report prediction time to improve the understanding of the feasibility of the proposed model.

This systematic review suggested a rapid advancement of research in this field with all included articles published between 2021 and 2023, reflecting the promising future of AI on TMD diagnosis. New AI approaches used in medicine have not yet been tested for this purpose. As an example, recent advances in AI gave rise to visual transformer networks which can learn global relationships from data.^28^ This can overcome the main limitation of CNNs of ignoring global pixel relationships within an image, limiting their generalization ability. Therefore, transformer hybridization with CNNs has been proposed as a method to enhance the model’s performance.^29–31^ Only 1 study discussed vision transformer but didn’t show any results related to it,^23^ therefore, this should be explored in future TMJ MRI models.

Overall, this systematic review demonstrated robust findings where all published studies consistently demonstrated high accuracy of AI for disk segmentation and displacement classification, at least in the single-centre datasets each examined. A high risk of bias related to the patient selection domain was identified and this was due to the case-control design and no information on inappropriate exclusions. However, the applicability concerns domain was assessed as low, in the absence of issues related to patient selection within centres that could affect the study question. The reference standard domain presented a high risk of bias in a few studies, due to the lack of information on the calibration process between the experts and how the diagnosis was made by the experts. Missing information in the calibration process might be an issue considering that poor agreement between MRI readers to assess TMJ has been reported in the literature.^3^^,^^4^ In addition, no information about how the experts classified the position of the disc in normal and displaced discs compromises the interpretation of the results related to the developed model’s performance. Regarding methodological quality, only the clustering domain presented major problems due to the lack of information. The missing information in some domains demonstrates that future studies should be better planned and reported and the use of checklists for AI in dental research or imaging is encouraged.

This systematic review has a few limitations. The high heterogeneity of the included studies prevented a meta-analysis; therefore, we were unable to provide a detailed quantitative synthesis of the model’s performance. Another limitation is that we excluded thesis and abstract proceedings from this review in this fast-moving field, and considered only English-language studies, which may exclude relevant studies that could have impacted our results.

## Conclusion

The integration of AI, particularly deep learning, in TMJ MRI, shows consistently promising results as a diagnostic-assistance tool to segment TMJ structures and to classify disc position, in primarily Asian single-centre studies. Further studies exploring more diverse multicentre data will improve the validity and generalizability of the models and are strongly recommended before any of these models are considered for implementation in clinical practice.

## Supplementary Material

twae055_Supplementary_Data
