# Supplementary material for: Temporomandibular joint assessment in MRI images using artificial intelligence tools: where are we now? A systematic review
Source: Dentomaxillofac Radiol. 2024 Nov 19;54(1):1–11. doi: 10.1093/dmfr/twae055 (PMC11800278; doi:10.1093/dmfr/twae055)
Supplement: twae055_Supplementary_Data [file twae055_supplementary_data.zip › twae055_Supplementary_Data/Appendix_1.docx]

Appendix S1. Search strategies with appropriate key words and MeSH terms.

| Database | Search strategy (Up to May 27^th^, 2024) |
| --- | --- |
| Embase | exp artificial intelligence/ or exp machine learning/ or computer assisted diagnosis/ or pattern recognition, automated/ OR artificial intelligence.mp.OR ((deep or machine) adj2 learning).mp.OR ((artificial or convolution*) adj2 neural network*).mp.  AND  exp Temporomandibular Joint Disorder/ OR ((temporomandibular joint or TMJ) adj2 (disorder* or dysfunction or disease*)).mp.  limit to (embase or "preprints (unpublished, non-peer reviewed)") |
| Medline | exp artificial intelligence/ or diagnosis, computer-assisted/ or pattern recognition, automated/ OR artificial intelligence.mp. OR ((deep or machine) adj2 learning).mp. OR ((artificial or convolution*) adj2 neural network*).mp.  AND  exp Temporomandibular Joint Disorders/ OR ((temporomandibular joint or TMJ) adj2 (disorder* or dysfunction or disease*)).mp. |
| Scopus | ( TITLE-ABS-KEY ( "temporomandibular joint" OR tmj W/2 disorder* OR dysfunction* OR disease* ) AND TITLE-ABS-KEY ( deep OR machine W/2 learning OR "artificial intelligence" ) OR TITLE-ABS-KEY ( artificial OR convolution* W/2 "neural network" OR "neural networks" ) OR TITLE-ABS-KEY ( computer W/2 assisted W/2 diagnosis OR pattern W/2 recognition ) ) |
| Web of Science | "artificial intelligence"  ((deep OR machine) NEAR/2 learning)  ((artificial OR convolution*) NEAR/2 "neural network*")  #1 OR #2 OR #3  (("temporomandibular joint" OR TMJ) NEAR/2 (disorder* OR dysfunction OR disease*))  #4 AND #5 |
| Google Scholar | “temporomandibular joint” OR tmj AROUND(2) (disorder* OR dysfunction*OR disease*) AND (deep OR machine AROUND(2) learning OR “artificial intelligence”) OR (artificial OR convolution* AROUND(2) “neural network” OR “neural networks”) OR ( computer AROUND(2) assisted AROUND(2) diagnosis OR pattern AROUND(2) recognition) site:.edu OR site:.org OR site.gov OR site:.ca OR site:.eu OR site:.uk OR site:.eu OR site:.au OR site:.nz |
