# Supplementary material for: Temporomandibular joint assessment in MRI images using artificial intelligence tools: where are we now? A systematic review
Source: Dentomaxillofac Radiol. 2024 Nov 19;54(1):1–11. doi: 10.1093/dmfr/twae055 (PMC11800278; doi:10.1093/dmfr/twae055)
Supplement: twae055_Supplementary_Data [file twae055_supplementary_data.zip › twae055_Supplementary_Data/Appendix_2.docx]

**Appendix S2.** Excluded articles and reasons for exclusion (n=38)

| **Author, Year** | **Reason for exclusion** |
| --- | --- |
| Bas et al 2012 | 1 |
| Becker et al 2018 | 1 |
| Belikova et al 2021 | 3 |
| Bianchi et al 2020 | 3 |
| Binaghi et al 2008 | 1 |
| Brosset et al 2020 | 3 |
| Davant et al 1993 | 2 |
| Dumast et al 2018 | 3 |
| Eser et al 2023 | 3 |
| Feifel et al 1991 | 2 |
| Ferrillo et al 2023 | 4 |
| Haghnegahdar et al 2018 | 3 |
| Hidaka et al 2023 | 1 |
| Isaieva et al 2023 | 2 |
| Iwasaki et al 2015 | 2 |
| Kim et al 2021 | 3 |
| Kordas et al 2015 | 5 |
| Kreiner et al 2022 | 1 |
| Le et al 2022 | 3 |
| Lee et al 2020 | 3 |
| Lee et al 2022 | 2 |
| Li et al 2021 | 4 |
| Liu et al 2021 | 3 |
| Mackie et al 2022 | 3 |
| McCartney et al 2014 | 5 |
| Mehta et al 2022 | 5 |
| Moura et al 2022 | 5 |
| Radke et al 2003 | 1 |
| Ribera et al 2019 | 3 |
| Shoukri et al 2019 | 3 |
| Unsal et al 2021 | 5 |
| Vinayahalingham et al 2023 | 3 |
| Wang et al 2021 | 3 |
| Warner et al 2022 | 3 |
| Yolacan et al 2022 | 5 |
| Zhang et al 2020 | 3 |
| Zhang et al 2021 | 3 |
| Zou et al 2022 | 1 |

1. Studies using clinical data but no imaging (n=7);
2. Studies in which AI was not used or it was used with another focus other than TMJ detection and diagnosis (n=5)
3. Studies training AI with other image modality other than MRI (n=18);
4. Studies with no reference standard (human labeling) as a comparison (n=2);
5. Reviews, personal opinions, abstracts, case reports, cases series, book chapters, letters (n=6)

REFERENCES

1. Bas, B.; Ozgonenel, O.; Ozden, B.; Bekcioglu, B.; Bulut, E.; Kurt, M. Use of artificial neural network in differentiation of subgroups of temporomandibular internal derangements: A preliminary study. Journal of Oral and Maxillofacial Surgery 2012;70(1):51-59.
2. Becker, Kai; Jakstat, Holger A; Ahlers, M Oliver. Quality improvement of functional diagnostics in dentistry through computer-aided diagnosis: a randomized controlled trial. International journal of computerized dentistry / 2018;21(4):281-294.
3. Belikova, Kristina; Zailer, Aleksandra; Tekucheva, Svetlana V; Ermoljev, Sergey N; Dylov, Dmitry V. Deep learning for Spatio-temporal localization of temporomandibular joint in ultrasound videos. 2021 IEEE International Conference on Bioinformatics and Biomedicine (BIBM) 2021;():1257-1261
4. Bianchi, J.; de Oliveira Ruellas, A.C.; Gonçalves, J.R.; Paniagua, B.; Prieto, J.C.; Styner, M.; Li, T.; Zhu, H.; Sugai, J.; Giannobile, W.; Benavides, E.; Soki, F.; Yatabe, M.; Ashman, L.; Walker, D.; Soroushmehr, R.; Najarian, K.; Cevidanes, L.H.S. Osteoarthritis of the Temporomandibular Joint can be diagnosed earlier using biomarkers and machine learning. Scientific Reports 2020;10(1):
5. Binaghi, Elisabetta; Gallo, Ignazio; Ghiselli, Cristina; Levrini, Luca; Biondi, Katia. An integrated fuzzy logic and web-based framework for active protocol support. International journal of medical informatics / 2008;77(4):256-71
6. Brosset, Serge; Dumont, Maxime; Bianchi, Jonas; Ruellas, Antonio; Cevidanes, Lucia; Yatabe, Marilia; Goncalves, Joao; Benavides, Erika; Soki, Fabiana; Paniagua, Beatriz; Prieto, Juan; Najarian, Kayvan; Gryak, Jonathan; Soroushmehr, Reza. 3D Auto-Segmentation of Mandibular Condyles. Annual International Conference of the IEEE Engineering in Medicine and Biology Society. IEEE Engineering in Medicine and Biology Society. Annual International Conference / 2020;2020(101763872):1270-1273
7. Davant, T S 6th; Greene, C S; Perry, H T; Lautenschlager, E P. A quantitative computer-assisted analysis of disc displacement in patients with internal derangement using sagittal view magnetic resonance imaging. Journal of oral and maxillofacial surgery : official journal of the American Association of Oral and Maxillofacial Surgeons / 1993;51(9):974-81.
8. de Dumast, P.; Mirabel, C.; Cevidanes, L.; Ruellas, A.; Yatabe, M.; Ioshida, M.; Ribera, N.T.; Michoud, L.; Gomes, L.; Huang, C.; Zhu, H.; Muniz, L.; Shoukri, B.; Paniagua, B.; Styner, M.; Pieper, S.; Budin, F.; Vimort, J.-B.; Pascal, L.; Prieto, J.C. A web-based system for neural network based classification in temporomandibular joint osteoarthritis. Computerized Medical Imaging and Graphics 2018;67():45-54.
9. Eşer, Gözde; Duman, Şuayip Burak; Bayrakdar, İbrahim Şevki; Çelik, Özer. Classification of Temporomandibular Joint Osteoarthritis on Cone‐Beam Computed Tomography Images Using Artificial Intelligence System. Journal of Oral Rehabilitation 2023;():
10. Feifel, H; Riediger, D. [TMJ diagnosis in oral and maxillofacial surgery]. Deutsche Zeitschrift fur Mund-, Kiefer- und Gesichts-Chirurgie / 1991;15(6):458-64
11. Ferrillo, M.; Migliario, M.; Marotta, N.; Fortunato, F.; Bindi, M.; Pezzotti, F.; Ammendolia, A.; Giudice, A.; Foglio Bonda, P.L.; de Sire, A. Temporomandibular disorders and neck pain in primary headache patients: a retrospective machine learning study. Acta Odontologica Scandinavica 2023;81(2):151-157.
12. Haghnegahdar, A.A.; Kolahi, S.; Khojastepour, L.; Tajeripour, F. Diagnosis of tempromandibular disorders using local binary patterns. Journal of Biomedical Physics and Engineering 2018;8(1):I-X
13. Hidaka T.; Tanaka K.; Mori H. An Artificial Intelligence-Based Cosmesis Evaluation for Temporomandibular Joint Reconstruction. Laryngoscope / 2023;133(4):841-848.
14. Isaieva, K.; Leclère, J.; Felblinger, J.; Gillet, R.; Dubernard, X.; Vuissoz, P.-A. Methodology for quantitative evaluation of mandibular condyles motion symmetricity from real-time MRI in the axial plane. Magnetic Resonance Imaging 2023;102():115-125.
15. Iwasaki, H.Bayesian belief network analysis applied to determine the progression of temporomandibular disorders using MRI. Dentomaxillofacial Radiology 2015;44(4):
16. Kim, Young Hyun; Shin, Jin Young; Lee, Ari; Park, Seungtae; Han, Sang-Sun; Hwang, Hyung Ju. Automated cortical thickness measurement of the mandibular condyle head on CBCT images using a deep learning method. Scientific Reports 2021;11(1):14852.
17. Kordas, Bernd; Ruge, Sebastian. On the analysis of condylar path versus real motion of the temporomandibular joint: application for Sicat Function. International journal of computerized dentistry / 2015;18(3):225-35.
18. Kreiner M, Viloria J. A novel artificial neural network for the diagnosis of orofacial pain and temporomandibular disorders. J Oral Rehabil. 2022;49:884- 889.
19. Le, Celia; Deleat-Besson, Romain; Prieto, Juan; Brosset, Serge; Dumont, Maxime; Zhang, Winston; Cevidanes, Lucia; Bianchi, Jonas; Ruellas, Antonio; Gomes, Liliane. Automatic segmentation of mandibular ramus and condyles. 2021 43rd Annual International Conference of the IEEE Engineering in Medicine & Biology Society (EMBC) 2021;():2952-2955.
20. Lee, K S; Kwak, H J; Oh, J M; Jha, N; Kim, Y J; Kim, W; Baik, U B; Ryu, J J.Automated Detection of TMJ Osteoarthritis Based on Artificial Intelligence. Journal of dental research / 2020;99(12):1363-1367.
21. Lee, C.; Ha, E.-G.; Choi, Y.J.; Jeon, K.J.; Han, S.-S. Synthesis of T2-weighted images from proton density images using a generative adversarial network in a temporomandibular joint magnetic resonance imaging protocol. Imaging Science in Dentistry 2022;52(4):393-408.
22. Li, Jupeng; Wang, Yinghui; Wang, Shuai; Zhang, Kai; Li, Gang. Landmark-guided rigid registration for temporomandibular joint MRI-CBCT images with large field-of-view difference. Machine Learning in Medical Imaging: 12th International Workshop, MLMI 2021, Held in Conjunction with MICCAI 2021, Strasbourg, France, September 27, 2021, Proceedings 12 2021;():527-536
23. Liu, Yi; Lu, Yao; Fan, Yubo; Mao, Longxia. Tracking-based deep learning method for temporomandibular joint segmentation. Annals of translational medicine / 2021;9(6):467.
24. Mackie, Tamara; Al Turkestani, Najla; Bianchi, Jonas; Li, Tengfei; Ruellas, Antonio; Gurgel, Marcela; Benavides, Erika; Soki, Fabiana; Cevidanes, Lucia. Quantitative bone imaging biomarkers and joint space analysis of the articular Fossa in temporomandibular joint osteoarthritis using artificial intelligence models. Frontiers in dental medicine 2022;3():1007011
25. McCartney, S.; Weltin, M.; Burchiel, K.J. Use of an artificial neural network for diagnosis of facial pain syndromes: An update. Stereotactic and Functional Neurosurgery 2014;92(1):44-52
26. Mehta, Anita; Mittal, Isham; Kakkar, Anushka. Artificial Intelligence in Dental Medicine. Acta Scientific Dental Sciences (ISSN: 2581-4893) 2022;6(2):2022
27. de Souza Moura, Brenda; Quesado, Gustavo; Ferreira, Natalia Reis; Oliveira, Aleli Tôrres; Grossmann, Eduardo; DaSilva, Alexandre F; DosSantos, Marcos Fabio. Accuracy of the Artificial Intelligence to Locate the Temporomandibular Disc in MRI. 2022; ():2022
28. John C. Radke, Robert Ketcham & Barry Glassman (2003) Artificial Neural Network Learns to Differentiate Normal TMJs and Nonreducing Displaced Disks after Training on Incisor-Point Chewing Movements, CRANIO®, 21:4, 259-264.
29. Ribera, N.T.; De Dumast, P.; Yatabe, M.; Ruellas, A.; Ioshida, M.; Paniagua, B.; Styner, M.; Gonçalves, J.R.; Bianchi, J.; Cevidanes, L.; Prieto, J.-C. Shape variation analyzer: A classifier for temporomandibular joint damaged by osteoarthritis. Progress in Biomedical Optics and Imaging - Proceedings of SPIE 2019;10950():2019.
30. Shoukri, B; Prieto, J C; Ruellas, A; Yatabe, M; Sugai, J; Styner, M; Zhu, H; Huang, C; Paniagua, B; Aronovich, S; Ashman, L; Benavides, E; de Dumast, P; Ribera, N T; Mirabel, C; Michoud, L; Allohaibi, Z; Ioshida, M; Bittencourt, L; Fattori, L; Gomes, L R; Cevidanes, L. Minimally Invasive Approach for Diagnosing TMJ Osteoarthritis.Journal of dental research / 2019;98(10):1103-1111.
31. Ünsal, Gürkan; Orhan, Kaan. Deep learning and artificial intelligence applications in dentomaxillofacial radiology. Applied Machine Learning and Multi-Criteria Decision-Making in Healthcare 2021;():124.
32. Vinayahalingam, S.; Berends, B.; Baan, F.; Moin, D.A.; van Luijn, R.; Bergé, S.; Xi, T. Deep learning for automated segmentation of the temporomandibular joint. Journal of Dentistry 2023;132():2023.
33. Wang, Shuai; Li, Jupeng; Peng, Yahui; Feng, Jiling; Ma, Ruohan; Li, Gang. Cone Beam CT Series Images Rigid Registration for Temporomandibular Joint via Self-supervised Learning Network. 2021 IEEE International Conference on Medical Imaging Physics and Engineering (ICMIPE) 2021;():1-5
34. Warner, E.; Al-Turkestani, N.; Bianchi, J.; Gurgel, M.L.; Cevidanes, L.; Rao, A. Predicting Osteoarthritis of the Temporomandibular Joint Using Random Forest with Privileged Information. Lecture Notes in Computer Science (including subseries Lecture Notes in Artificial Intelligence and Lecture Notes in Bioinformatics) 2022;13755 LNCS():77-86.
35. Yolaçan, Ebru; Demircan, Nimet Ebrar; Ercikti, Nurcan; Kocabiyik, Necdet; Yalçin, Bülent. TEMPOROMANDIBULAR JOINT AND ARTIFICIAL INTELLIGENCE. Romanian Journal of Functional & Clinical, Macro-& Microscopical Anatomy & of Anthropology 2022;21(2):
36. Zhang, Kai; Li, Jupeng; Ma, Ruohan; Li, Gang. An end-to-end segmentation network for the temporomandibular joints CBCT image based on 3D U-Net. 2020 13th International Congress on Image and Signal Processing, BioMedical Engineering and Informatics (CISP-BMEI) 2020;():664-668.
37. Zhang, Kai; Li, Jupeng; Ma, Ruohan; Li, Gang. 3D Temporomandibular Joint CBCT Image Segmentation via Multi-directional Resampling Ensemble Learning Network. Machine Learning in Medical Imaging: 12th International Workshop, MLMI 2021, Strasbourg, France, September 27, 2021, Proceedings 12 2021;634-643.
38. Zou, Wei; Mao, Bomin; Fadlullah, Zubair Md; Qi, Kun. Assisting in Diagnosis of Temporomandibular Disorders: A Deep Learning Approach. IEEE Access 2022;10():124076-124082.
