# Supplementary material for: Temporomandibular joint assessment in MRI images using artificial intelligence tools: where are we now? A systematic review
Source: Dentomaxillofac Radiol. 2024 Nov 19;54(1):1–11. doi: 10.1093/dmfr/twae055 (PMC11800278; doi:10.1093/dmfr/twae055)
Supplement: twae055_Supplementary_Data [file twae055_supplementary_data.zip › twae055_Supplementary_Data/Appendix_3.docx]

**Appendix 4.** Analysis of the studies included on the AI model. Assessed by the checklist for Ai in dental research proposed Schwendicke et. al

| **Study (author,year)** | **Sampling** | **Processing** | **Protection** | **Sample Size** | **Reference test** | **Clustering** | **Dataset test** | **Computational resource** | **Total** |
| --- | --- | --- | --- | --- | --- | --- | --- | --- | --- |
| Bai et al 2023 | 1 | 1 | 0 | 1 | 1 | 0 | 1 | 1 | 6/8 |
| Ito et al 2022 | 1 | 1 | 0 | 1 | 1 | 0 | 1 | 1 | 6/8 |
| Kao et al 2023 | 1 | 1 | 1 | 1 | 1 | 0 | 1 | 1 | 7/8 |
| Kim et al 2021 | 1 | 1 | 1 | 0 | 1 | 0 | 1 | 1 | 6/8 |
| Lee et al 2022 | 1 | 1 | 0 | 1 | 1 | 0 | 1 | 1 | 6/8 |
| Li et al 2022 | 1 | 1 | 1 | 1 | 1 | 0 | 1 | 1 | 7/8 |
| Lin et al 2022 | 1 | 1 | 1 | 0 | 1 | 0 | 1 | 1 | 6/8 |
| Nozawa et al 2022 | 1 | 1 | 0 | 1 | 1 | 0 | 1 | 1 | 6/8 |
| Orhan et al 2021 | 1 | 1 | 1 | 1 | 1 | 1 | 1 | 1 | 8/8 |
| Oszari et al 2023 | 1 | 1 | 0 | 1 | 1 | 0 | 1 | 1 | 6/8 |
| Wu et al 2022 | 0 | 1 | 0 | 0 | 1 | 0 | 1 | 1 | 4/8 |
| Yoon et al 2023 | 1 | 1 | 1 | 1 | 1 | 0 | 1 | 1 | 7/8 |
| Yoshimi et al 2023 | 1 | 1 | 0 | 1 | 1 | 0 | 1 | 1 | 6/8 |

Absent = 0; present = 1
